# Applying a sustainability perspective in the literature on physical therapy in relation to pharmaceuticals: a scoping review

**DOI:** 10.3389/fpubh.2024.1509677

**Published:** 2025-01-07

**Authors:** Patric Svensson, Magdalena Jacobsson, Annie Palstam, Elvira Lange

**Affiliations:** ^1^School of Health and Welfare, Dalarna University, Dalarna, Sweden; ^2^Rehabilitation Medicine, Institute of Neuroscience and Physiology, Department of Clinical Neuroscience Sahlgrenska Academy, University of Gothenburg, Gothenburg, Sweden; ^3^Department of Neurology, Sahlgrenska University Hospital, Gothenburg, Sweden; ^4^Department of General Practice, Institute of Medicine at the Sahlgrenska Academy, University of Gothenburg, Gothenburg, Sweden; ^5^Research, Education, Development and Innovation, Primary Health Care, Region Västra Götaland, Gothenburg, Sweden

**Keywords:** physical therapy, physiotherapy, sustainable development, triple bottom line, scoping approach

## Abstract

**Introduction:**

Physical therapy encompasses a broad range of treatment options, often utilized in clinical settings where pharmaceutical interventions are standard. The potential for physical therapy to contribute to sustainable healthcare by reducing environmental impact, while maintaining the quality of care, remains underexplored. This study aimed to map existing research comparing physical therapy to pharmaceuticals, with a specific focus on whether these studies address aspects of sustainable development.

**Methods:**

A scoping review was conducted, systematically searching the PubMed, Cinahl, and Pedro databases using keywords related to physical therapy, pharmaceuticals, and comparative studies. Two assessors independently reviewed and selected relevant studies, followed by data extraction and summarization of results.

**Results:**

A total of 27 studies were included, varying in design, population, and healthcare context. The most commonly addressed conditions were osteoarthritis and musculoskeletal pain, with analgesics being the most frequently studied pharmaceutical interventions. While several studies touched upon economic and social dimensions of sustainable development, none examined environmental sustainability. This highlights a critical gap in current research.

**Discussion:**

Future studies are needed to assess how physical therapy, when compared to pharmaceutical treatments, can contribute to sustainable healthcare by offering a low-carbon, resource-efficient alternative without compromising social sustainability through adverse effects. This knowledge could be instrumental in guiding healthcare systems toward more sustainable practices.

**Systematic review registration:**

A study protocol was registered in Open Science Framework 2023-03-31 (Available from: https://osf.io/we58g).

## Introduction

Healthcare accounts for a significant share of global greenhouse gas emissions, i.e., carbon footprint, and the largest share is due to the production, transport and consumption of goods and services such as pharmaceuticals and other chemicals, foods and medical equipment (1, 2). Thus, healthcare has a responsibility to actively participate in the transition towards sustainable development (2), defined as ‘development that meets the needs of the present without compromising the ability of future generations to meet their own needs’, involving economic, environmental and social sustainability (3). A sustainable healthcare builds on the three dimensions of sustainable development (2, 4), and is defined as a system that maintains and improves the health of current generations while minimizing damage to the environment and ensuring health for future generations (4). Four key elements have been identified to enable sustainable healthcare (5): disease prevention and health promotion to reduce the need for healthcare; self-management and empowerment of patients to take a greater role in managing their own health and healthcare; lean service delivery and; prioritizing low carbon alternatives (2).

The three dimensions of sustainable development are further described in the business framework the Triple Bottom Line (6). This framework argues that for a business to be sustainable, it needs to consider not only the economic aspect, but also the environmental and social aspects of operations. In other words, it must consider for example air pollution and greenhouse gas emissions, and the health and wellbeing of employees and clients (6). Triple Bottom Line has since its’ introduction been employed in studies (2, 4, 7). The Triple Bottom Line has been found particularly interesting in healthcare since health is directly and indirectly determined by ecological and social aspects (8).

From the perspective of physical therapy (PT), individuals have inherent resources to maintain or improve their health on their own or with the facilitation of others (9). In PT, support for behavioral change, maintenance and resumption of activities, function and abilities, and the promotion of patient empowerment are central concepts (7). The concept of empowerment is based on the idea that people themselves have the ability and resources to define their own problems and devise action strategies to deal with them. Behavior change is an important part of health promotion and disease prevention (10), together with patient education and rehabilitation (10). The fact that PT is a treatment option without the direct environmental impact of, for example, pharmaceuticals (11) implies PT is key for sustainable healthcare.

Pharmaceutical care is the responsible provision of pharmaceuticals (e.g., drugs, medicines) for the purpose of achieving outcomes that improve a patient’s quality of life (12). Pharmaceuticals affect physiological processes in the body, usually by binding to various proteins (13). Pharmaceuticals are usually chemically stable substances, allowing them to withstand a certain passage through the body before they reach their area of action (13). These properties and ways of acting in pharmaceuticals are primarily intended to promote treatment outcomes, but they can also have negative effects through various adverse effects in the user as well as a negative impact on the environment (13).

The adverse effects of pharmaceuticals can vary in severity and depend, among other things, on the substance used, who is using them, and in what dose (3). Side effects of pharmaceuticals have a major impact on society, leading to high costs, increased morbidity requiring care, and risk of death (12). The production and consumption of pharmaceuticals contribute to a global problem of pollution in water and soil (5, 6). These pollutants affect human health and the lives of plants and animals and have been identified as major problems in several countries (14–16). The groups of pharmaceuticals most abundant in the environment are analgesics, specifically Non-steroidal Anti-Inflammatory Drugs (NSAIDs) (17).

The use of pharmaceuticals leads to negative environmental impact and possible negative side effects for the individual. PT as a treatment has been shown to have an effect in several areas where pharmaceuticals are commonly used, such as high blood pressure, depression, diabetes, and osteoarthritis (11, 18). However, there is a lack of collective knowledge about the areas in which PT could influence pharmaceutical use. This is an important area, as this knowledge could contribute the transition to sustainable healthcare. The aim of this study was to investigate what research, in form of scientific articles, is available that study PT in relation to pharmaceuticals. The aim was also to investigate whether this research relates their results to sustainable development regarding environmental-, social- or economic aspects.

## Methods

### Study design

This study utilized a scoping review methodology to investigate the extent and type of available research. The methodological framework proposed by Arksey and O’Malley (19) was followed, with some additions from Levac et al. (20). A study protocol was registered in Open Science Framework 2023-03-31 (Available from: https://osf.io/we58g) and the PRISMA ScR Checklist was used when reporting the results (21).

### Research questions

The research questions guiding this scoping review were formulated as follows:

1. What research exists that compares PT and pharmaceutical treatments, or that assesses pharmaceutical use/prescription as outcomes from PT?
2. To what extent are aspects of sustainable development addressed, in articles on PT in relation to pharmaceuticals?

### Literature search

A comprehensive literature search was conducted on 2023-02-20 to identify relevant studies. PubMed, Cinahl, and PEDro databases were searched using combinations of keywords related to PT, pharmaceuticals, and comparative studies, with no limitation on the date (Table 1). The search strategy was adjusted based on sample searches, and consultations with a librarian, to optimize search results.

**Table 1:** Overview of the search strategy.

	Search	Hits
PubMed	(physical therapy modalities OR physical therapy specialty OR physiotherapy) AND (pharmaceutical preparations OR pharmaceutical intervention OR drugs) AND (comparative study) NOT (animals OR animal experimentation)	771
Cinahl	(physical therapy modalities OR physical therapy specialty OR physiotherapy) AND (pharmaceutical preparations OR pharmaceutical intervention OR drugs) AND (comparative study) NOT (animals OR animal experimentation)	229
PEDro	physi* pharmaceutical* compar*	63
	physi* drugs* compar*	191

### Study selection

Based on the following inclusion and exclusion criteria, studies were selected from the search result:

*Inclusion Criteria:*

- Original articles and study protocols that in some way compare PT and pharmaceuticals as treatment options, with PT explicitly mentioned as PT by the authors, or interventions performed or supervised by a physical therapist, or performed in a PT clinic.
- Studies that evaluate pharmaceutical consumption as an outcome measure via either intake or via prescription, or by evaluating a pharmaceutical treatment as an intervention.

*Exclusion Criteria:*

- Articles involving animal studies.
- Articles not available in English.

The selection process was performed by two assessors independently (PS and MJ), with the help of Rayyan.ai software, and started with screening of titles and abstracts. Relevant articles were further assessed by reading their full texts. Inclusion and exclusion criteria were applied individually by the authors. The selection was based on agreement and discrepancies were identified and resolved through discussion within the author group, until consensus was reached.

### Data charting

Data from the included studies were extracted and organized into a table. The collected information included author details, year of publication, study location, study design, intervention type, study population, research purpose, methodology, outcome measures, significant results, and aspects of sustainable development.

### Collating, summarizing, and reporting the results

The included articles were analyzed and presented in relation to the research questions, in terms of their characteristics, physical therapy interventions, types of pharmaceuticals, pharmaceutical consumption as an outcome measure, and discussions related to sustainable development. The scoping review aimed to provide an overview of the available research rather than conducting a synthesis of the evidence.

## Results

**Figure 1:**
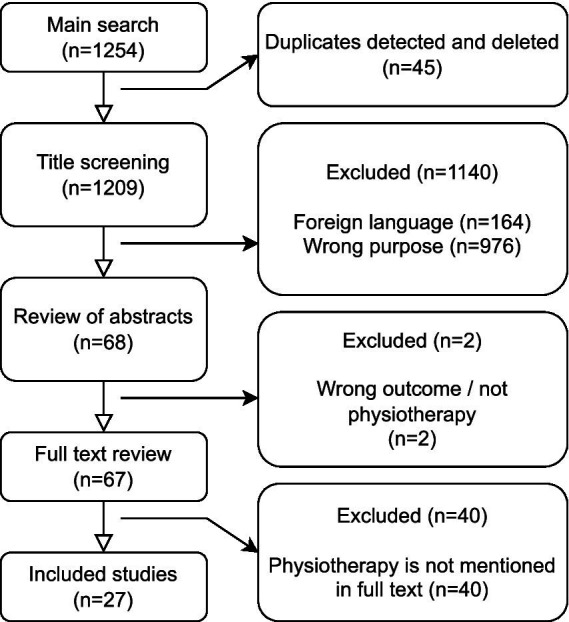
Flow chart of main search and inclusion process.

The search in PubMed, Cinahl and PEDro resulted in 1254 articles (Figure 1) of which 45 duplicates were detected and deleted, leaving 1,209 unique articles for title screening. Out of 1,209 articles, 68 were selected for further review of abstract after the title screening based on the study’s inclusion- and exclusion criterium. The abstracts were then reviewed, and 66 studies were selected for full-text review. Finally, 27 articles (22–48) were included after the study selection process, the most common reason for studies not being included was that they did not address PT explicitly as treatment (Figure 1).

The included studies (22–48) were published between 1998 and 2022 and conducted in 15 different countries, with the most studies conducted in Turkey (23, 37, 39–41, 47, 48) and the USA (24, 25, 34, 46) (Table 2). Most of the studies (17 out of 27) were randomized controlled trials (23, 24, 28–31, 35, 39–42, 44–48), and another four had a randomized design (26, 27, 37, 38), three of the studies where retrospective (25, 34, 36), one was a prospective observational study (43), one was an open-label alternate patient treatment allocation study (33) and two were study protocols (27, 32). The most common diagnoses in the included studies were osteoarthritis (OA), back pain and shoulder pain. The other studies commonly included musculoskeletal diagnoses, but also respiratory and gynecological diagnoses.

**Table 2:** Characteristics of included studies.

Author, year, country, study design	Objectives	Population (age, diagnosis, sex, number)	Physical therapy	Pharmaceuticals	Outcomes	Overall results	Sustainable development perspective
Uygur, E. et al. (2019), Turkey (48) Randomized controlled trial	To compare dry needling with cortisone injection as treatment for plantar fasciitis.	96 patients with plantar fasciitis that required continued treatment after 3 weeks of initial treatment. 66% female. Mean age ≈ 49.6.	Dry needling in the plantar fascia 2 times per week on 5 occasions.	Corticosteroid containing: Methylprednisolone acetate; Bupivacaine. 1 injection into the plantar fascia.	Pain, Proms function	Positive effects in favor of dry needling, when compared with injections after 6 months.	Environmental: no Economic: yes Social: no
Deyle, Gail D. et al. (2020), USA (46) Randomized controlled trial	To examine differences in treatment effectiveness for pain and physical function in the short and long term regarding injection and PT.	156 patients with knee OA. 48% female. Mean age ≈ 56.1.	Passive joint mobilization and strength training ≤8 sessions over 4–6 weeks +1–3 sessions at the time of the 4-month and 9-month reassessments	Glucocorticoid steroids containing: Triamcinolone acetonide; Lidocaine. 1–3 injections.	Proms function, Health economics, Functional tests	Positive effects on pain and physical function in favor of PT, when compared to injections after 12 months.	Environmental: no Economic: yes Social: no
Bakilan, F. & Ortanca, B. (2021), Turkey (37) Non-randomized controlled trial	To compare the patient satisfaction and pain relief firstly between patients who received pharmacological treatment and non-pharmacological treatment.	109 patients with chronic low back pain. 65.14% female. Mean age ≈ 47.9.	Hotpack; Therapeutical ultrasound; TENS	NSAID; Myorelaxant; Lidocaine	Pain, Proms satisfaction	Positive effects on pain and satisfaction in favor of PT, when compared to pharmaceutical care.	Environmental: no Economic: yes Social: no
Bisset, L. et al. 2006, Australia (22) Randomized controlled trial	To investigate the efficacy of PT compared with a wait and see approach or corticosteroid injections over 52 weeks in tennis elbow.	198 adults with a clinical diagnosis of tennis elbow ≥6 weeks, who had not received any other active treatment by a health practitioner in the previous 6 months. 35% female. Mean age ≈ 47.6.	8 sessions of elbow manipulation and therapeutic exercises over 6 weeks + home exercise with resistance exercise band. Patient education to all participants.	Corticosteroid containing: Lidocaine; Triaminolone acetonide. 1–2 injections. Patient education to all participants.	Pain, Proms function, Function tests, Assessor rating of severity	Positive effects in favor of CSI, compared with PT after 6 weeks. Positive effects in favor of PT at long time assessment when compared to CSI.	Environmental: no Economic: no Social: no
Hamzat, Talhatu K. et al. (2011), Nigeria (38) Quasi-experimental study	To determine the respective and combined effects of NSAIDs and PT in management of pain and reduced functional limitation among patients with concurrent hypertension and knee OA.	33 patients with both high blood pressure and knee OA of which 29 completed the study. 65.52% female. Mean age ≈ 65.7.	TENS; Strength training 3 times per week 8 weeks treatment period.	Anti-hypertensive drugs; NSAID 8 weeks treatment period.	Pain, Proms function	No significant differences between groups.	Environmental: no Economic: yes Social: yes
el Refaye, G. E. et al. (2019), Egypt (45) Randomized, single-blind, controlled trial	To determine which is more effective in alleviating primary dysmenorrhea: PEMF or diclofenac drugs.	50 females with regular menstruation. Mean age ≈ 21.88.	PEMF on the pelvic region. 3 occasions per menstrual cycle during 3 cycles	Diclofenac when in pain during 3 menstrual cycles.	Pain, Blood test, Proms symptoms	Positive effects on all outcomes, in favor of PT, when compared to pharmaceuticals.	Environmental: no Economic: no Social: no
Gür, A. et al. (2002), Turkey (47) Randomized, single-blind, placebo-controlled study	To examine the effectiveness of low power laser and low-dose amitriptyline therapy and to investigate effects of these therapy modalities on clinical symptoms and QoL in patients with fibromyalgia.	75 patients with fibromyalgia. 80% female. Mean age ≈ 30.	Laser therapy. Every weekday for 2 weeks.	Amitriptyline daily for 8 weeks.	Pain, pain assessment, proms symptoms, proms QoL and mental health	Positive effects on pain and fatigue, in favor of PT, when compared to pharmaceuticals. Positive effects on mental health, in favor of pharmaceuticals, when compared to PT.	Environmental: no Economic: no Social: yes
Koc, Z. et al. (2009), Turkey (39) Randomized, single-blind, controlled trial	To compare the effects of epidural steroid injections and PT on pain and function in patients with LSS.	29 patients with lumbar spinal stenosis. 72.41% female. Mean age ≈ 59.1.	Ultrasound; Hot pack; TENS 5 times per week for 2 weeks; All participants: home-exercise for 6 months, diclofenac for 2 weeks.	Steroid containing: Triamcinolon acetonide; Bupivacaine hydrochloride; Physiologic saline. Epidural injection. All participants: home-exercise for 6 months, diclofenac for 2 weeks.	Pain, functional tests	No significant differences between groups.	Environmental: no Economic: no Social: no
Atamaz, F. et al. (2006), Turkey (40) Randomized, single-blind study	To compare the effects of PT and two different intra-articular hyaluronan drugs (sodium hyaluronate (NaHA) and hylan G-F 20) on knee OA.	82 patients with knee OA. 81.71% female. Mean age ≈ 60.	Infrared; Short-wave diathermy pulsed patterns; Interferential therapy.	NaHA containing: 15 mg Sodium hyaluronate and 9 mg Sodium chloride. Hylan G-F 20 containing: 8 mg HA, 0.16 mg Sodium chloride and 0.04 mg Sodium dihydrogen phosphate hydrate. Intraarticular injection.	Pain, functional tests, Proms function, Proms QoL	Positive effects on pain and QoL, in favor of PT, when compared to injections. Positive effects on mental health, in favor of Injections, when compared to PT.	Environmental: no Economic: yes Social: yes
Paker, N. et al. (2006), Turkey Prospective Randomized Study	To investigate the efficacy of TENS and of intra-articular hylan G-F 20 injection in terms of pain, functional status, and QoL parameters in patients with knee OA, and to monitor effects of treatment over a 26-week follow-up period.	52 patients with knee OA. No information regarding gender. Mean age ≈ 58.92.	TENS 20 min 5 times per week for 3 weeks.	Hylan G-F 20 3 intraarticular knee injections once per week.	Pain, Functional tests, Proms function, Proms QoL	No significant differences between groups.	Environmental: no Economic: no Social: no
Sadeghifar, Amirreza et al. (2022), Iran (36) Retrospective comparative study	To evaluate the efficacy of Subacromial CSI, HA injection and PT in patients with SIS to determine which treatment is most effective.	88 patients with subacromial impingement syndrome. 53.41% female. Mean age ≈ 43.	Stretching exercises; Specific strength training for shoulder muscles; Hot pack; TENS; Ultrasound. 3 sessions per week for 3 weeks followed by home exercises for 1 month	Triamcinolone; HA 2 CSI with 2 week interval.	Pain, Proms function	Positive effects, in favor of CSI, when compared to PT at 3 and 6 months.	Environmental: no Economic: no Social: no
Arslan, S. & Celiker, R (2001), Turkey (23) Randomized controlled trial	To compare the efficacy of local steroid injection and PT measures for treating adhesive capsulitis.	20 patients with shoulder pain and limited ROM. 50% female. Mean age ≈ 56.	Hot pack; Ultrasonic therapy; Passive glenohumeral joint stretching exercises; Specific shoulder exercises such as wall climbing	NSAID; Steroid containing: Methylprednisolone acetate. Local CSI.	Pain, ROM	No significant differences between groups.	Environmental: no Economic: no Social: no
Naikmasur, V. G. et al. (2009), India (42) Randomized controlled trial	To assess the effectiveness of PT methods in myofascial pain patients and to compare with pharmacotherapy comprising of a combination of muscles relaxants and NSAID.	40 patients with myofascial pain of masticatory muscles. 57% female. Mean age ≈ 34.76.	TENS; Ultrasound; Helium-neon laser	Ibuprofen; Paracetamol; Chlorzoxazone. 2 times per day for 5 days	Pain, pain assessment, Functional tests, Proms function	Positive effects on pain and function, in favor of PT, when compared to pharmaceutical care.	Environmental: no Economic: yes Social: yes
Lahti, M. et al. (2008), New Zealand (26) Pilot randomized trial.	To assess the ease of the day-to-day running and to review the implementation of the trial protocol. The secondary objective for the pilot study was to compare the treatments, that is, the primary objective for the main study.	57 female with predominant urge urinary incontinence experiencing at least monthly leakage. Mean age ≈ 55.	Bladder retraining therapy containing: Patient information and education; Pelvic floor muscle training. 3 months.	Anticholinergic drug: Oxybutynin; Tolterodin 3 months.	Proms QoL, Proms symptoms	No significant differences between groups.	Environmental: no Economic: yes Social: yes
Galán-Martín, Miguel A. et al. (2019), Spain (27) Study protocol for a randomized, multicenter clinical trial	To evaluate the effectiveness and cost-effectiveness of a program based on active coping strategies that include Pain Neuroscience Education and physical exercise by means of a randomized clinical trial compared to usual care in primary care PT.	Patients with non-specific back pain of ≥6 months. Age 18–70 years.	Pain neuroscience education program in 6 sessions (10 h) + group exercise 18 sessions led by physical therapist; Usual primary care PT consisting of 15 sessions of analgesic electrotherapy, thermotherapy and standardized physical exercise.	-	Pain, pain assessment, Proms QoL, Proms symptoms, Satisfaction, Pharmaceutical use.; Health Services visits;	Not applicable (study protocol)	Environmental: no Economic: yes Social: yes
Henderson, J. et al. (2020), Australia (43) A prospective observational study	To identify if a collaborative care ED PCP after-hours service would result in improvements in ED treatment times for musculoskeletal and simple orthopedic presentations compared with patients managed under a secondary contact model of care. Secondary aims to identify if there were differences in orthopedic, follow-up plans on discharge, and analgesia prescriptions.	1,640 patients in ED setting. No information regarding gender. Age ≥ 16 years.	Primary or secondary contact for patients in ED	Analgesics	Mean treatment time, orthopedic or GP referrals, Prescribed analgesics	Positive effects on referrals, prescriptions and early discharge, in favor of primary contact PT, when compared to secondary contact PT.	Environmental: no Economic: yes Social: no
den Hertog, A. et al. (2012), Germany (28) Randomized prospective clinical study	To investigate fast-track rehabilitation concept in terms of a measurable effect on the early recovery after TKA.	147 patients that had underwent TKA surgery. 70.75% female. Mean age ≈ 67.	Fast-track rehabilitation: Group therapy; Early mobilization and individual PT. Standard rehabilitation: Individual postoperative care according to existing protocol.	Analgesics	Proms function, Length of hospital stay, Consumption of analgesics	Positive effects on function, Consumption of analgesics, and length of stay, in favor of Fast-track rehabilitation, when compared to standard rehabilitation.	Environmental: no Economic: yes Social: no
Rhon, Daniel I. et al. (2014), USA (24) Randomized controlled trial	To compare the effectiveness of 2 common non-surgical treatments for SIS.	98 patients with subacromial impingement syndrome. 31.63% female. Mean age ≈ 41.	Joint and soft-tissue mobilizations; Manual stretches; Contract-relax techniques; Reinforcing exercises directed to the shoulder girdle or thoracic/cervical spine	Triamcinolone acetonide. CSI.	Pain, Proms function, Shoulder-related health care use, Number of additional injections	Positive effects on health care use, in favor of PT, when compared to CSI. No significant differences between groups in pain and function.	Environmental: no Economic: yes Social: no
Brose, Steven W. et al. (2019), USA (25) Retrospective analysis	To determine the success of a multidisciplinary project to manage chronic pain while reducing reliance on opioids in a population of patients with spinal cord injury.	Individuals with spinal chord injury receiving outpatient care.	Exercises.	Opioids	Opioid prescription rate; Number of patients receiving opioids; Equivalent morphine quantity	Positive effects on opioid prescription rate and number of patients receiving opioids, in favor or the multidisciplinary project, compared to previous care.	Environmental: no Economic: no Social: yes
Gallefoss, F. (2004), Norway (35) Randomized controlled trial	To explore the effects and health economic consequences of patient education in patients with COPD in a 12-month follow-up.	62 patients with mild to moderate COPD. 50% female. Mean age ≈ 58.	Patient education regarding breathing and coughing, exercising and management of attacks.	Beta2-agonist inhalation; Ipratropium bromide; Salbutamol	Number of GP consultations; Proportions in need of GP consultations; Utilization of rescue pharmaceuticals; Patient satisfaction	Positive effects on GP consultations, in favor of patient education, when compared to a group with no education.	Environmental: no Economic: yes Social: no
van Baar, M. E. et al. (1998), Netherlands (30) Randomized, single-blind, clinical trial	To determine the effectiveness of exercise therapy in patients with OA of hip or knee.	201 patients with osteoarthritis of the hip or knee. 78.11% female. Mean age ≈ 68.	Exercise therapy from physical therapist. 12 weeks	NSAID; Paracetamol	Pain, Functional tests, Proms effect, ROM, Pharmaceutical use.	Positive effects on pain, function and pharmaceutical use, in favor of PT, when compared to treatment as usual.	Environmental: no Economic: no Social: no
van Baar, M. E. et al. (2001), Netherlands (29) Randomized, single-blind, clinical trial	To determine whether the effects of exercise therapy in patients with OA of hip or knee are sustained after 6 and 9 months’ follow up.	183 patients with osteoarthritis of the hip or knee. 85.79% female. Mean age ≈ 68.	Exercise therapy from physical therapist. 12 weeks	NSAID; Paracetamol	Pain, Functional tests, Proms effect, ROM, Pharmaceutical use.	Positive effects on pain, in favor of PT, was found at 24 weeks, but no differences remained at 36 weeks.	Environmental: no Economic: no Social: no
Hay, E. M. et al. (2006), UK (31) Pragmatic multicenter randomized clinical trial.	To compare the clinical effectiveness, in primary care, of enhanced pharmacy review or community PT with that of a control intervention (advice leaflet reinforced by atelephone call) in the treatment of adults aged 55 years and over consulting their GP with knee pain.	325 adults with knee pain. 64.33% female. Mean age ≈ 67.7.	Patient education about the safety and importance of exercise, pacing, pain relief, and coping strategies; Individualized exercise program.	NSAID; Analgesics	Pain, Proms function	Self-reported use of NSAID and simple analgesia was significantly lower in the PT group compared to the control group. For the pharmacy group, the use of NSAID was significantly lower and the use of simple analgesia significantly higher compared to the control group.	Environmental: no Economic: yes Social: yes
Clausen, B. et al. (2014), Denmark (32) Study protocol for a randomized, single-blind, controlled trial	To compare the efficacy of a specific neuromuscular exercise program with optimized analgesics and anti-inflammatory drug use on knee loads, as well as pain and physical function in people with mild to moderate medial tibiofemoral knee OA.	100 patients with knee OA. Age 40–70 years.	Exercises to improve balance, muscle activation, functional alignment, and functional joint stability.	NSAID; Acetaminophen	Pain, Functional tests, QoL, Pharmaceutical use.	Not applicable (study protocol)	Environmental: no Economic: yes Social: yes
Joshi, M. N. et al. (2009), India (33) Open-label alternate patient treatment allocation	To compare the performance of PT and amitriptyline for disability reduction in patients with fibromyalgia. To determine which clinical features at baseline would predict benefit with either therapy.	156 outpatients with fibromyalgia syndrome. 96.15% female. Mean age ≈ 39.	Specific exercises; Relaxation; Stretching; Strength training	Amitriptyline; Tramadol	Proms function, Pharmaceutical use (tramadol).	No significant differences between groups.	Environmental: no Economic: yes Social: yes
Gagnon, R. et al. (2021), Canada (44) Randomized controlled trial	To compare the effects of direct-access PT to usual care provided by an emergency physician for patients presenting to the ED with a MSKD.	78 patients with minor MSKD. 44% female. Mean age ≈ 40.2.	Direct access to physical therapist in the ED including: Physical examination; Patient education; Interventions based on the clinical analysis and physical therapists diagnosis (technical aids, imaging, prescribed or over-the-counter medication, consults with other health care professionals)	Opioids; Over-the-counter drugs	Pain, Utilization of services and resources at ED; Health care professionals consulted; Imaging tests recommended	Positive effects on clinical outcomes and utilization of services and resources, in Favor of the direct access PT, when compared to usual care after discharge and 3 months later.	Environmental: no Economic: yes Social: yes
Kim, Howard S. et al. (2019), USA (34) Retrospective cohort study	To compare analgesic prescribing among ED visits for back or neck pain receiving PT versus usual care.	464 patients with back pain visiting ED. 59% female. Mean age ≈ 48.2.	Physical examination of patient; Guidance on activity progression; Home exercises; Patient information and education	Benzodiazepine; Other opioid drugs	Pain; Prescription of opioids or benzodiazepine at discharge	ED back and neck pain visits receiving PT were no less likely to receive an opioid prescription and were more likely to receive a benzodiazepine than visits receiving usual care.	Environmental: no Economic: yes Social: yes

OA, osteoarthritis; TKA, Total knee arthroplasty; ROM, Range of motion; COPD, Chronic obstructional pulmonary disease; ED, Emergency department; MSKD, Musculoskeletal Disorders; LSS, Lumbar spinal stenosis; SIS, Subacromial impingement Syndrome; PCP, Primary Care Provider; NSAID, Non-Steroid Anti-inflammatory Drug; PT, Physical therapy; PEMF, Pulsed Electromagnetic Field Therapy; TENS, Transcutan Electronic Nerve Stimulation; HA, Hyaluronic acid; GP, General Practitioner; Proms, Patient reported outcome measures; QoL, Quality of Life; BMI, Body Mass Index; FIQ, Fibromyalgia Impact Questionnaire; WOMAC, The Western Ontario and McMaster Universities Arthritis Index; VAS, Visual Analog Scale; SPADI, Shoulder Pain and Disability Index; GRC, Global Rating of Change; NPRS, Numeric Pain Rating Scale; CSI, Corticosteroid injections; AKSS, American Knee Society Score; HRQoL, Health-related Quality of Life; SF-12, 12-Item Short Form Survey; SF-36, 36-Item Short Form Survey.

In the included studies, many different types of PT interventions were described, comprising various exercise interventions (20 of 27) (22–36, 38, 42–44, 46) and interventions where the patient passively received treatment (15 of 27) (22–24, 27, 36–42, 45–48) (Table 2). Other interventions found in the included studies were patient education (22, 26, 27, 31, 34, 35) and organizational health care changes (Table 2) (34, 43, 44).

Of the pharmaceuticals that appeared in the studies, the most common type was analgesics; primary NSAIDs, followed by cortisone or other injections, muscle relaxants, and pharmaceuticals for depression and anxiety. Other pharmaceuticals that occurred were blood pressure lowering substances, anti-choleric, and pharmaceuticals to facilitate breathing (Table 2).

Twelve of the studies (24, 25, 28–35, 43, 44) had pharmaceutical use- or prescription as an specified outcome measure. Of these studies, nine compared PT with treatment as usual (25, 27–30, 34, 35, 43, 44), one study compared PT with pharmaceutical review (31) and two studies compared PT with pharmaceutical interventions (32, 33). Of the studies that compared PT with usual treatment, three (34, 43) compared PT at the ED with either no-PT ED care or PT at a later stage. Two studies (29, 30) compared PT with standard treatment by a physician, one with standard treatment by physical therapist (27), one compared fast-track rehabilitation with rehabilitation according to routine (28), and two studies (25, 35) compared multimodal therapy and educational programs containing PT with treatment as usual and routine PT.

Fifteen studies compared PT interventions, without pharmaceutical treatment, with pharmaceutical interventions but did not specify pharmaceutical use as a direct outcome measure (22, 23, 26, 36–42, 45–48). In one of these studies the participants still reported their pharmaceutical intake, which was presented in the results (22). Of the 15 studies, nine compared PT with some type of injection (22–24, 36, 39–41, 46, 48) (cortisone, hyaluronic, NaHa, or steroid). The types of pharmaceuticals in the other six studies were: anticholinergics (26), NSAIDs or other analgesics (37, 38, 42, 45), muscle relaxants (37, 42), blood pressure lowering pharmaceuticals (38) and antidepressants (47).

None of the included articles discussed sustainable development, and none performed evaluations from a Triple Bottom Line since environmental aspects of sustainable development were not addressed in any study. Eight of the studies did not address any aspect of sustainable development (22, 23, 29, 30, 36, 39, 41, 45). Economic aspects of sustainable development related to costs and healthcare utilization, were discussed in 17 of the included studies (24, 26–28, 31–35, 37, 38, 40, 42–44, 46, 48). Six of those studies compared costs and utilization between their intervention and control groups (2, 24, 27, 28, 35, 44). One study (26) suggests for future studies that cost analyses should be carried out. Social aspects of sustainable development were addressed in 12 of the included studies (25–27, 31–34, 38, 40, 42, 44, 47). One study (33) addresses poverty, education level and unequal access to care. Quality of life or physical and mental health were included as outcome measures in eight studies (26, 27, 31–33, 40, 44, 47), but they were not discussed from a sustainable development perspective. Three studies discussed patient abilities and active participation in care (31, 42, 44) and two discussed risks to patients, such as overmedication (25, 34). In one study, TENS was suggested as an alternative for pain management if pharmaceutical treatments where contraindicated (38). Negative effects of prolonged NSAID use, and the positive effects on coping through active patient engagement in PT were highlighted (42). Empowerment and self-management was discussed as possible reasons for lowered use of health care services and resources in PT treatment (44).

## Discussion

This scoping review found that investigations on PT in relation to pharmaceuticals as treatment hold a variation of characteristics with different study designs, study samples and in a range of areas within healthcare. The perspective of sustainable development was not applied in any of the included studies, and environmental aspects were not considered at all. However, several studies did consider economic or social sustainability to some extent.

Among the 27 included studies, many different types of interventions were described as PT. Physical exercise in different forms, was the most frequent type of PT treatment in the studies and TENS was the second most frequent form of PT intervention. In most of the included articles PT was referred to as specific rehabilitation exercises (22–36), in some it was referred to as TENS (36–43) and, in one study, PT was referred to as electromagnetic field treatment (45). These examples mirror the diversity of interventions within the PT discipline, highlighting the importance to mention PT in the title, abstract or keywords when addressing an intervention within the wide scope of PT, when applicable. Nevertheless, to describe an intervention solely as ‘physical therapy’ would not be of sufficient detail (49). Thus, we argue that a clear description of the intervention, including if it is delivered within the PT discipline is necessary to facilitate systematic study of interventions delivered within the PT profession.

None of the included studies were found to address all three dimensions of sustainable development, i.e., the Triple Bottom Line. That absence of evaluations regarding environmental sustainability in any study, can be considered a missed opportunity as the high carbon footprint and pollution from pharmaceuticals is a well-known problem where an effective PT alternative could pose an important contribution to sustainable healthcare. The environmental impact of PT on for example facilities and water use should not be ignored, but could be considered minor compared to the total emissions from health care, especially when compared to pharmaceuticals (2). However, some studies did discuss economic and social aspects of sustainable development. Economic evaluations mainly concerned costs and utilization of health care resources where evaluations of social aspects involved patient participation and empowerment as means for reduced healthcare utilization over time. The importance of patient empowerment for sustainable healthcare has been recognized by the WHO and is proposed by the Centre for Sustainable Healthcare as a cornerstone for sustainable healthcare (2, 4). Another aspect of sustainability that was addressed in the included studies was over medication (25) and the negative consequences from side effects were discussed in another (38). In line with this, the inconvenience of side effects from pharmaceuticals compared with PT interventions has been described previously in research of pain management (50), and patients have previously been found to prefer non-pharmacological pain management over opioids as treatment (51). Hence, increasing societal knowledge and awareness of PT as a non-pharmacological, non-invasive pain treatment is essential (52). Evaluation from the triple bottom line has been suggested in PT research (53). For such evaluations, cooperation between physical therapists, environmental scientists and economists could be of great value for assuring that all parts of the triple bottom line are considered in a holistic perspective on sustainable healthcare.

A strength of our study is the systematic methodology and following the stepwise process described by Arksey and O’malley with additions by Levac et al. (19, 20). Combining these two when conducting a scoping review is recommended for a structured methodology (54). The choice of study design was appropriate to be able to chart in which areas comparisons of PT and pharmaceuticals exist, in order to address gaps in knowledge and identify areas where systematic reviews would be possible. There have also been at least two reviewers of all articles at all stages. Making the selection of studies in a team is something recommended by Levac et al. (20).

One limitation of this study is that grey literature was not included. The primary reason we only searched databases is because we were mainly interested in scientific sources. This could have led to publications bias and a possible lack of included studies with non-significant results. Another limitation is only including articles in English, which may have meant that we missed interesting research that could have contributed to our results; the reduction in linguistic and cultural variation could have led to lower generalizability. The result could also be limited due to the choice to only include three databases in the search strategy, as well as the specific choice of databases. Further, the decision to only include studies that used certain terminology concerning PT, it was noted during the exclusion process that many potentially interesting studies were to be excluded. By only including studies where PT was mentioned explicitly, many studies that involved exercise interventions, TENS, acupuncture, and other interventions that may well be referred to as PT were excluded. This is problematic since similar interventions would be stated as PT in the included studies but were excluded due to the lack of mentioning of PT. However, it would not have been appropriate to define PT by naming all possible interventions, considering the wide range shown already in this study. Therefore, we emphasize the importance of carefully consider terminology in studies evaluating PT interventions, to facilitate future systematic research in the field of PT.

## Conclusion

There is a need for further research on the role of PT interventions in relation to pharmaceuticals as treatment. Studies are lacking that consider sustainable development, and studies comparing PT and pharmaceuticals that evaluate outcomes from a perspective of sustainable development could contribute with knowledge about how PT can be a low-carbon, resource efficient alternative to pharmaceuticals, without their negative impact on social sustainability in terms of adverse effects, and thus contribute to sustainable healthcare.

## Data Availability

The original contributions presented in the study are included in the article, further inquiries can be directed to the corresponding author.
